# The effect of the immune system on ovarian function and features of ovarian germline stem cells

**DOI:** 10.1186/s40064-016-2390-3

**Published:** 2016-07-07

**Authors:** Haifeng Ye, Xiaoyan Li, Tuochen Zheng, Xia Liang, Jia Li, Jian Huang, Zezheng Pan, Yuehui Zheng

**Affiliations:** School of Life Science, Nanchang University, Nanchang, China; Medical Teaching Laboratory Center, Jiangxi Medical College, Nanchang University, Nanchang, China; The Key Laboratory of Reproductive Physiology and Pathology of Jiangxi Province, Nanchang, China; Faculty of Basic Medical Science, Jiangxi Medical College, Nanchang University, Nanchang, China; School of the 1st Clinical Medical Sciences, Jiangxi Medical College, Nanchang University, Nanchang, China

**Keywords:** Immune system, Ovarian function, Ovarian germline stem cells

## Abstract

In addition to its role in maintaining organism homeostasis, the immune system also plays a crucial role in the modulation of ovarian function, as it regulates ovarian development, follicular maturation, ovulation and the formation of the corpus luteum. Ovarian germline stem cells are pluripotent stem cells derived from the ovarian cortex that can differentiate into ovarian germ cells and primary granulosa cells. Recent work has demonstrated that the proliferation and differentiation of ovarian germline stem cells is regulated in part by immune cells and their secreted factors. This paper reviews the role of the immune system in the regulation of ovarian function, the relationship between immune components and ovarian germline stem cells and current research efforts in this field.

## The immune system maintains tissue homeostasis

### Introduction to the immune system

The immune system, which plays an important role in immune responses and immunologic function, consists of immune organs (including bone marrow, thymus and lymph nodes), immune cells (including lymphocytes, monocytes and neutrophils) and secreted factors such as serum complement proteins, immunoglobulins, interferons and tumour necrosis factor. The immune system is subdivided into several groups. The first division is between innate (non-specific) and adaptive (specific) immunity, and adaptive immunity can be further divided into humoral and cell-mediated immunity depending on the target antigen. The immune system is critical for resisting pathogen invasion and has three vital functions: (1) immune defence, or combatting the invasion of pathogenic microorganisms such as viruses, bacteria and other contaminants; (2) immunological surveillance, or recognizing, killing and eliminating mutated cells to prevent cancers; and (3) immunological homeostasis, or removing aged and dying cells to maintain bodily homeostasis. The intersection of these three functions is the key to the maintenance of homeostasis, whereas immune dysfunction can promote disease.

### The contribution of the immune system to tissue homeostasis and the maintenance of tissue-self-state

Most cells in the body do not directly contact the external environment but rather exist and survive in an internal environment consisting of extracellular fluid such as plasma, interstitial fluid, lymph or cerebrospinal fluid (CSF). Maintenance of the internal environment is crucial for bodily homeostasis. In addition to their role in defence, recent work has shown that the cells and molecules of the immune system can regulate the proliferation, differentiation and apoptosis of cells in several tissues, thereby helping to maintain internal environment homeostasis. Carrel (1922) found that leukocyte infusion (a liquid analogous to tissue extracts) could stimulate the proliferation of fibroblasts in vitro, after which he postulated that leukocytes were a source of growth factors. Later work demonstrated that lymphocytes also could promote tissue development and regeneration. For example, Fidler (1980) suggested that lymphocytes could regulate tissue development by acting as trophoblasts. In recent years, monocyte-derive cells (MDCs) have been shown to play a crucial role in tissue maintenance, and cytokines and growth factors derived from this cell type can also influence cellular differentiation and function (Bukovsky et al. 2000). Furthermore, scientists have also found that the immune system not only regulates the balance of the internal environment but also plays an important role in the maintenance of tissue steady state.

Available data indicate that the functions of all tissues are regulated via ‘morphostasis’, a process whereby tissues retain a specific differentiation status after the organism completes morphogenesis. This process is essential to maintain the homeostasis of the internal environment. ‘Morphostasis’ is a complex process that is completed in three steps during embryonic development. The first step involves the renewal of tissue stem cells, while in the second step, specific cells in tissue can be conserved in proper differentiation status. The final step regulates tissue amount and is carried out by the tissue control system (TCS) in which the immune system plays an important role (Bukovsky 2011).

The TCS is mainly composed of cells from the immune and autonomic nervous system, as well as vascular endothelial cells (Bukovsky et al. 1983, 1991, 1995). The functions of these cell types are established early during embryonic development. In addition, MDCs, T lymphocytes and B lymphocytes also possess crucial functions with respect to the TCS (Bukovsky 2011). According to the prevailing hypothesis in this field, MDCs in the TCS can send out ‘stop effects’ to prevent cells from further differentiation after their respective fates have been specified. Next, autonomic innervation controls the number of volume of cells and tissues, respectively, thus maintaining “morphostasis”. Importantly, this step relies upon ‘stop effects’ from MDCs (Bukovsky et al. 2000). Importantly, the TCS can regulate specific organs and tissues of the body and plays a crucial role in the regeneration of ovarian follicles and age-related anovulation (Bukovsky 2007).

With increasing age, degeneration of the immune system and reduced ‘stop effects’ from MDCs can alter the “morphostasis” of many organs, including ovaries, and result in functional declines (Bukovsky 2007).

## The role of the immune system in ovary

### The relationship between the immune system and ovary

Since the 1970s, scientists have made great strides in understanding the relationship between the immune system and the ovaries. For example, Sakakura and Nishizuka (1972) found that the ovaries of athymic mice failed to develop within 2–4 days after birth. In addition, Russell et al. (1973) used thymocytes from wild type female mice to suppress cyclophosphamide- and X-ray-induced super ovulation. Furthermore, Bukovsky and Presl (1979) postulated that the immune system is a crucial mediator of ovarian function, a hypothesis supported by recent research. For example, studies have shown that thymosin injections in neonatal nude mice can help maintain the levels of follicle stimulating hormone (FSH) and luteinizing hormone (LH) during post-natal development. Therefore, this method can rescue aberrant ovarian development in nude mice (Goya et al. 2007). In addition, studies have also shown that the immune system can degenerate with age, followed by reductions in ovarian function (Bukovsky and Caudle 2012). This correlation indicates the close relationship between the immune system and ovary.

### The interaction between the immune system and ovarian endocrine function

It is well established that the hypothalamic-pituitary axis is crucial for proper ovarian function. Under the influence of hormones from the hypothalamus, the hypophysis secretes FSH and LH, which underlie the physiological changes associated with the female menstrual cycle. In addition, various immune cells exist within the tissues of the hypothalamus–pituitary–ovarian axis, indicating that the immune system may regulate ovarian function and degeneration. Indeed, in the early 1980s and 1990s, studies of the relationship between immunity and the ovary showed that immune cell-derived cytokines can modulate the production of hormones along the hypothalamus–pituitary–ovary axis, thereby indicating that the immune system can influence reproductive physiology.

During ovarian follicle maturation, increasing pressure in the follicular cavity causes the follicle to rupture, leading to the release of a secondary oocyte surrounded by the zona pelludica and corona radiata. This process is called ovulation, and cytokines play an important role in both early and late stages follicular development (Crespo et al. 2010). For example, granulosa cells in the follicles produce tumour necrosis factor α (TNF-α), which promotes the secretion of FSH. These cytokines can also inhibit prostaglandin F2a (PGF2a), which in turn, facilitates ovulation (Nakao et al. 2015). Studies in mice have further shown that ovarian TNF-α can prevent FSH from stimulating aromatase activity and progestin secretion, thereby promoting ovulation (Nakao et al. 2015). In addition, interleukin-1 (IL-1) can reduce the expression of LH receptors in granulosa cells, as well as suppress progestin secretion. Moreover, IL-1 also facilitates the progression of ovulation and oocyte development (Gerard et al. 2004). Furthermore, FSH can synergize with transforming growth factor-β (TGF-β) to inhibit follicular development, thereby regulating the quantity of primordial follicles (Wang et al. 2014).

The corpus luteum produced during the menstrual cycle in mammals has a remarkably short life cycle. If the egg is not fertilized, the corpus luteum will regress into the corpus albicans within 2 weeks. Studies of many animal models have postulated that macrophage-derived secretions (e.g., TNF) participate in both the development and degeneration of corpus luteum (Galvao et al. 2013). Specifically, TNF can stimulate the secretion of vascular endothelial growth factor (VEGF) in the immature and developing corpus luteum, thereby stimulating vasculogenesis and the subsequent development of the mature corpus luteum (Galvao et al. 2013). TNF is also found in the degenerated corpus luteum. Specifically, TNF in the immature corpus luteum promotes its growth, while high levels in an unfertilized corpus luteum assist the progression of its apoptosis and degeneration. This dual role of TNF still remains a mystery (Galvao et al. 2013).

## Immunity and the proliferation and differentiation of germline stem cells

### Ovarian germline stem cells (OSCs)

The origins of the primordial follicles and oocytes in the ovaries of mature mammals have been debated for several hundreds of years. According to the views of traditional medical, most mammals are born with oocytes, which explains the limited supply of oocytes with respect to age. However, researchers in the field of phylogenetics hold a different view (Bukovsky et al. 2005). Specifically, Johnson et al. (2004) proposed that gametes possess mitotic activity and can self-renew, even in the mature ovary of adult mammals. Since that time, research on ovarian germline stem cells (OSC) has attracted more attention.

OSCs are epithelial cells that reside on the ovarian surface. Several molecular markers, including c-kit, Oct-4, vasa and telomerase have been detected on the ovarian surface epithelium (OSE) in many mammalian species (Bukovsky et al. 2008; Virant-Klun 2015; Bhartiya et al. 2013; Gheorghisan-Galateanu et al. 2014). Therefore, OSCs are also referred to as the ‘germline epithelium’.

In recent years, scientists have made considerable progress in understanding OSCs. For example, Zou et al. (2009) isolated germline stem cells both from new-born and adult mice, introduced a GFP expression construct and implanted the cells into sterile mice. These cells differentiated into functional follicles, and the sterile mice ultimately gave birth to GFP-expressing progeny. In addition, White et al. (2012) isolated germline stem cells from healthy individuals and injected them into both human ovaries and immunocompromised mice. Surprisingly, both procedures resulted in the formation of primordial follicles. Based on these results, the consensus in the field indicates that germline stem cells do exist in the ovary from birth. The stem cells can replenish the original primordial follicle pool via self-renewal and differentiation. Work in our laboratory seeks to determine the mechanism of OSC function.

### Immunity is a part of OSC niche

OSCs can differentiate into oocytes and granulosa cells, and they originate from the bipotential stem cells of the ovarian cortex (Li et al. 2015). This process is facilitated by features of the OSC niche. Specifically, Bukovsky (2011) postulated that factors within the OSC niche control OSC functions. Moreover, the OSC niche contains immune cells and their secreted molecules. The OSC niche is established during early stages of foetal development and consists of committed ovarian MDCs, T cells and vascular endothelial cells. By contrast, the adult OSC niche contains primary MDCs (CD14+ MDC), activated MDCs ([HLA-DR]+ MDC) and T cells. Therefore, immunity plays an important role in proliferation and differentiation of germline stem cells.

Bukovsky (2007) demonstrated that immune cells and their secreted factors in the OSC niche modulate the asymmetric cell division of OSCs. Ultimately, this process leads to the production of oocytes, and regulates the symmetric division, migration and generation of new granulosa cells. Immune cells also play an important role in the development of primordial follicles in both the foetus and adult, and support ovarian homeostasis. These conclusions are based on the following: ① According to studies of human ovarian development, follicles develop from the inner layer of the ovarian cortex adjacent to the rete ovarii. Importantly, this structure is required for follicular development (Byskov et al. 1977; Hummitzsch et al. 2015) and contains several immune cell types, such as CD14+ MDCs. These cells can differentiate into activated MDCs ([HLA -DR]+), which then migrate in reticular passageways and interact with innate MDCs. In addition, inhibitory T cells (CD3+ T cells) and cytotoxic T cells(CD8+ T) also reside in the reticular tubes. ② When OSCs differentiate into reproductive cells, they must then accept ovary-committed bone marrow cells (OCMT), which in turn, stimulate MDCs and T cells (Bukovsky 2015). OSCs can produce one differentiated germ cell and one progeny OSC. The germ cell can then differentiate into an oocyte and migrate to the epithelial layer adjacent to blood vessels in the ovarian cortex. As a consequence of normal circulatory function, germ cells will develop and interact with granulosa cells to form primitive follicles (Kossowska-Tomaszczuk and De Geyter 2013). Ultimately, the development and migration of germ cells occur in the context of immune cells such as CD14+ MDCs and require asymmetric OSC division. Moreover, symmetric germ cell division is accompanied by CD8+ T cells and is assisted by primary MDCs. Ultimately, primary MDCs facilitate differentiation of germ cells to epithelial cells, while activated MDCs (DR+ MDC) are involved in germ cell migration.

### Immunity establishes an ‘ovarian memory’

**Fig. 1:**
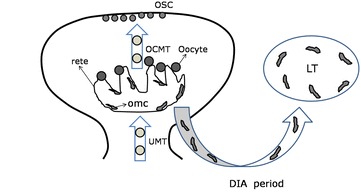
Generation of ovarian memory cells during developmental immune adaptation. *UMT* uncommitted MDC and T cells, *OCMT* ovary-committed bone marrow cells, *OMC* ovarian memory cells, *OSC* ovarian germline stem cells, *LT* lymphoid tissue, *DIA* developmental immune adaptation

**Fig. 2:**
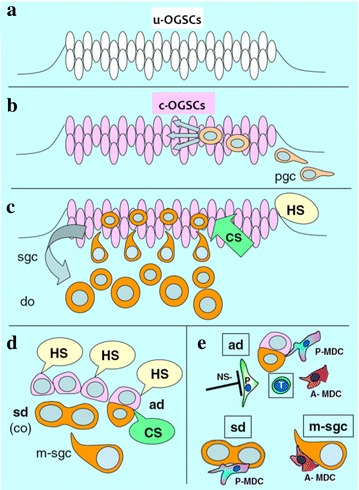
The role of the immune system in promoting the symmetric and asymmetric division and differentiation of ovarian germ stem cells. **a** Uncommitted OGSCs(u-OGSCs) were produced in the first six weeks of pregnancy; **b** Primordial germ cells (PGC) were invaded OGSCs layerin the first seven weeks of pregnancy; **c–e** OGSCs only the joint action of cell signaling (cellular signaling, CS; as CD14 secreted by MDC and CD8 secreted by T lymphocytes) and hormonal signals (hormonal signaling, HS; chorionic gonadotropin, hCG and estradiol, E2) can occur secondary asymmetric division to produce germ cells, germ cells into secondary ovarian cortex and eventually differentiate into oocytes (definitive oocytes, DO). Modified from Bukovsky and Caudle (2012)

Bukovsky (2011) has hypothesized that uncommitted MDC and T cells (UMT) can recognize and “memorize” OSC in the OSC niche. Bukovsky (2006) further hypothesized concerning ‘ovarian memory’: When primitive germ-line cells are implanted into undifferentiated gonads, the rete ovarii will stimulate the differentiation of secondary gonocytes to oocytes. During the development of adaptive immunity, many uncommitted MDCs and T cells (UMT) are present. Some of these UMT can differentiate into “veiled cells” in the channels of the rete ovarii and can transmit chemical messages from oocytes in the rete ovarii to developing lymph tissue. From this point on, these UMT are known as ovarian memory cells (OMC) (Fig. 1), which promote the differentiation of UMT in the channels of the rete ovarii to ovary-committed bone marrow cells (OCMT). These OCMT can then migrate to the ovarian epithelium, where they produce molecular signals to trigger both asymmetric and symmetric OSC division (Fig. 2). Thus, secondary germ-line cells are then produced. The development of secondary germ-line cells also relies on a suitable hormonal environment consisting of hCG and estradiol. According to this hypothesis, the developing adaptive immune system can establish an ‘ovarian memory’ during the foetal period to support the replenishment of OCMT in adulthood. This immune function will decline by the age of 35–40, concomitant with the replacement of follicles (Bukovsky et al. 2004, 2009).

As the development of the adaptive immune system nears completion, the foetal rete ovarii will undergo degeneration, thereby preventing further oogenesis. This process is caused by reductions in hormonal signals (the foetal hCG barrier) (Bukovsky et al. 2005). Further development of OMCs in the lymph nodes will then cease, and these cells will ultimately constitute the ovarian memory cell pool. During menarche and the reproductive years, hormonal signals and OMCT can facilitate periodic oogenesis by stimulating OSCs. Continuous renewal of follicles requires cyclic OCMT supplementation, as the memory cell pool will become exhausted if the OCMT proliferation rate in lymph tissue is too high. Therefore, even in the presence of hormone signals, depletions in the frequencies of OMCs will still terminate oogenesis. According to this view, as age increases, immunological anaplasia is the main point at which oogenesis and ovarian replenishment cease. This proposal is supported by extensive experimental data that reveal the close correlation between mammalian immunity and reproduction. This work has raised new questions about the influence of immunologically relevant molecules on the proliferation and differentiation of ovarian germline stem cells.

### Clinical application of immune system in regulating ovarian germline stem cells

The research of OSCs will eventually apply to clinical therapy, and the research has shown that there is a great value of clinical application in immune regulation OSCs. In vitro, autologous OSCs in vitro are expected to achieve the in vitro maturation and in vitro fertilization of infertile women (Bukovsky 2015). However, due to the lack of similar immune condition, OSCs cannot complete the meiotic differentiation of eggs in vitro. The research indicated that OSCs successfully differentiated into eggs by the co-culture of OSCs and mononuclear cells in vitro (Bukovsky et al. 2006), further experiments showed that cell differentiation and the new egg production were obvious by the culture of ovarian cortical cells that stemmed from the premenopausal and postmenopausal women in vitro. Undoubtedly, the above researches provided a theoretical and experimental support for the new in vitro fertilization technology.

In vivo, the our laboratory is trying to promote OSCs proliferation, differentiation and further remodel ovarian function during pathological and physiological ovarian aging by enhance the body immune function. Fortunately, these unpublished results show that the immune function and reproductive function can be improved synchronously through the treatment of phase-immune enhancement agents during premature ovarian failure in mice. Similarly, the results are expected to implement enhanced reproductive function in the body.

## Conclusion and prospects

This view of OSC function is distinct from the views of traditional reproductive medicine and reproductive biology. As germline cells self-renew in the ovaries of foetuses and adults, OSCs can maintain ovarian homeostasis. Thus, the study of cultured OSCs in vitro and in vivo may have clinical applications in the treatment of POF and ovarian infertility, and may improve pregnancy rates and postpone female ageing. In recent years, ovarian germline stem cells have been shown to reverse infertility in mouse models, but the exact mechanism of this phenomenon regulation is still not well understood. OSCs are influenced by many hormonal signals cell signalling factors. This review focused on the influence of immune cells and molecules to ovarian function, and considered the possibility that immunity controls the proliferation and differentiation of OSCs.

The immune system plays important roles both in evading foreign pathogens and maintaining tissue homeostasis. In adulthood, the functions of different tissues (including ovaries) all require mechanisms to regulate (1) stem cell renewal, (2) differentiation status and (3) tissue amount. The TSC is particularly important for the establishment of tissue steady states and also plays an important role in the immune system. Morphostasis takes place in the immune adaptive period, which is a key period in embryonic development. With increasing age, the immune system will undergo degenerative change, also leading to ovarian degeneration. In addition, recent studies indicate that the asymmetric division of OSC into new germline cells requires the stimulation MDCs and T cells. Moreover, immunologically relevant cells and their secretion also modulate the symmetric division of germline cells, as well as their symmetric division, migration and production of new granulosa cells and primitive follicles to maintain homeostasis in the ovary.

Scientists have made great strides in understanding immunity, reproduction and mechanisms of in vivo germline stem cell function. However, there is still considerable debate regarding the existence of germ-line stem cells. The isolation and purification of ovarian germline stem cells have only been accomplished in in vitro studies. However, further research on the proliferation and differentiation of ovarian germline stem cells may have important clinical applications, as it may help treat ovarian insufficiency, ovarian infertility and POF. It may also be a mechanism by which women can delay ageing.
